# Positive *vs.* Negative Facts

**Published:** 1897-10

**Authors:** M. R. Leverson

**Affiliations:** Fort Hamilton, L. I.


### POSITIVE *VS*. NEGATIVE FACTS.

[Translated from *Le Médecin*, official organ of the Medical school of Belgium, August 1st, 1897.]

M. R. LEVERSON, M. D., FORT HAMILTON, L. I.

It is an axiom in science that a thousand negative facts are worth less than one single well verified positive fact.

How, then, has it happened that in medical statistics more weight has been accorded to a procedure, or to a method, applied in conjunction with other methods directed to the same end, than to the failures of this same procedure or of this method *applied by itself* with exclusion of all other methods, as, for instance, in cases of croup, cholera, hydrophobia, the plague, etc.? How is it, too, that people continue to boast the advantages of the Jennerian vaccine (of the nature of syphilis), when it is proved that thousands of people "successfully vaccinated" (with the *success* that is to say of producing the chancre) at the age of two years have nevertheless fallen victims to small-pox at five or six?

And these positive facts accumulate, and still fail to crush the imbecile scaffolding of the negative ones!

A woman contracted small-pox. She had three children not yet syphilized by the Jennerians. The doctors insist that the children be forthwith vaccinated. The sick mother yields. Two children are subjected to the pestilential operation, when the father arrives and forbids the vaccination of the third. A month later the mother is convalescent and thoroughly cured of small-pox. The two vaccinated children catch the small-pox *and die of it.* The unvaccinated child did not get it, and enjoys good health.

Day after day thousands of similar positive facts happen upon the globe in the very faces of the vaccinators who cannot see them, but persist in pretending that their negative facts alone deserve to be collected and noticed.

